# Supplementary material for: Molecular Susceptibility and Treatment Challenges in Melanoma
Source: Cells. 2024 Aug 20;13(16):1383. doi: 10.3390/cells13161383 (PMC11352263; doi:10.3390/cells13161383)
Supplement: Supplementary file 1 [file cells-13-01383-s001.zip › cells-3141839-supplementary.pdf]

Supplementary document

Molecular Susceptibility and Treatment Challenges in Melanoma

Kiran Kumar Kolathur [1, †], Radhakanta Nag [2, †], Prathvi V Shenoy [3], Yagya Malik[3], Sai Manasa Varanasi [4], Ramcharan Angom [4] and Debabrata Mukhopadhyay [4]

Figure S1

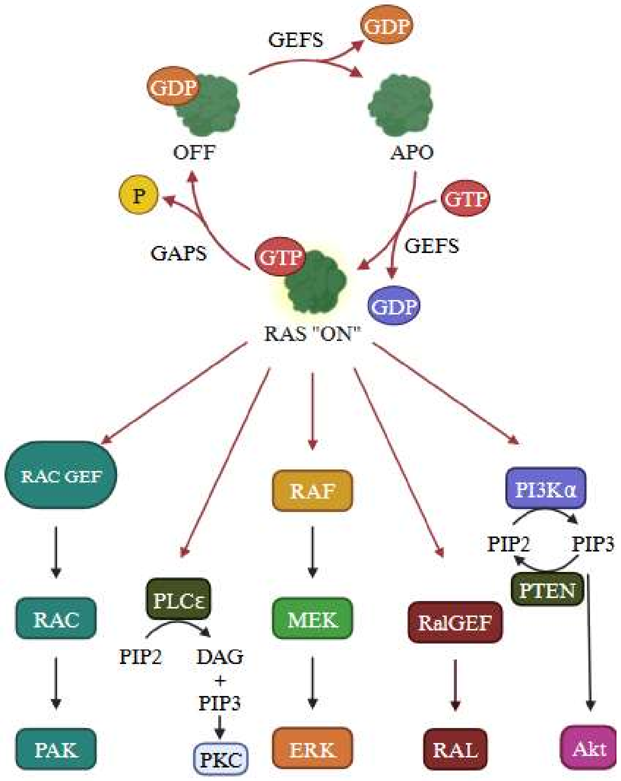

Figure S1. The figure depicts the downstream effect of the RAS signaling pathway diagram. The downstream effects of the RAS signaling pathway involving Ras-related C3 botulinum toxin substrate (RAC), p21 protein (Cdc42/Rac)-activated kinase (PAK), proto-oncogene serine/threonine-protein kinase (RAF), Ras-related protein Ral (RAL), Phosphatidylinositol 3,4,5-trisphosphate 3-phosphatase and dual-specificity protein phosphatase (PTEN), and RAC-alpha serine/threonine-protein kinase (AKT) can impact all fundamental processes of living cells. RAS cycles between inactive guanosine diphosphate (GDP)-bound and active GTP-bound states. This transition is mediated by guanine nucleotide exchange factors (GEFs) that promote the release of GDP to form a transient nucleotide-free state (apo RAS). Due to the picomolar affinity of apo RAS for nucleotide coupled with the higher concentration of GTP than GDP in cells, apo RAS subsequently binds GTP, leading to the activation of downstream effector pathways. Termination of RAS activation occurs upon hydrolysis of RAS-bound GTP, which is facilitated by the action of GTPase accelerating proteins (GAPs) that enhance the intrinsic GTPase activity. RAS regulates its downstream effectors by directly binding them through a single 'effector binding domain', which causes an elaborate control of effector binding by competition, binding affinities, abundances, and subcellular localization. The downstream signaling effects of the RAS pathway can lead to the activation of other MAP kinase family members (Created with BioRender.com).
